# Systematic Investigation of DNA Methylation Associated With Platinum Chemotherapy Resistance Across 13 Cancer Types

**DOI:** 10.3389/fphar.2021.616529

**Published:** 2021-04-29

**Authors:** Ruizheng Sun, Chao Du, Jiaxin Li, Yanhong Zhou, Wei Xiong, Juanjuan Xiang, Jiheng Liu, Zhigang Xiao, Li Fang, Zheng Li

**Affiliations:** ^1^ NHC Key Laboratory of Carcinogenesis, Hunan Cancer Hospital and the Affiliated Cancer Hospital of Xiangya School of Medicine, Central South University, Changsha, China; ^2^ The Key Laboratory of Carcinogenesis and Cancer Invasion of the Chinese Ministry of Education, Central South University, Changsha, China; ^3^ Department of Hematology and Oncology, The First Hospital of Changsha, Changsha, China; ^4^ Department of General Surgery, Hunan Provincial People’s Hospital, The First Affiliated Hospital of Hunan Normal University, Changsha, China

**Keywords:** methylation, DNA, drug resistance, neoplasm, platinum

## Abstract

**Background:** Platinum resistance poses a significant problem for oncology clinicians. As a result, the role of epigenetics and DNA methylation in platinum-based chemoresistance has gained increasing attention from researchers in recent years. A systematic investigation of aberrant methylation patterns related to platinum resistance across various cancer types is urgently needed.

**Methods:** We analyzed the platinum chemotherapy response-related methylation patterns from different perspectives of 618 patients across 13 cancer types and integrated transcriptional and clinical data. Spearman’s test was used to evaluate the correlation between methylation and gene expression. Cox analysis, the Kaplan-Meier method, and log-rank tests were performed to identify potential risk biomarkers based on differentially methylated positions (DMPs) and compare survival based on DMP values. Support vector machines and receiver operating characteristic curves were used to identify the platinum-response predictive DMPs.

**Results:** A total of 3,703 DMPs (*p* value < 0.001 and absolute delta beta >0.10) were identified, and the DMP numbers of each cancer type varied. A total of 39.83% of DMPs were hypermethylated and 60.17% were hypomethylated in platinum-resistant patients. Among them, 405 DMPs (Benjamini and Hochberg adjusted *p* value < 0.05) were found to be associated with prognosis in tumor patients treated with platinum-based regimens, and 664 DMPs displayed the potential to predict platinum chemotherapy response. In addition, we defined six DNA DMPs consisting of four gene members (mesothelin, protein kinase cAMP-dependent type II regulatory subunit beta, msh homeobox 1, and par-6 family cell polarity regulator alpha) that may have favorable prognostic and predictive values for platinum chemotherapy.

**Conclusion:** The methylation-transcription axis exists and participates in the complex biological mechanism of platinum resistance in various cancers. Six DMPs and four associated genes may have the potential to serve as promising epigenetic biomarkers for platinum-based chemotherapy and guide clinical selection of optimal treatment.

## Introduction

Platinum-based chemotherapy has been an indispensable anticancer therapy for nearly every cancer, but especially for urogenital, lung, and gastrointestinal cancers (Verhoeven et al., 2013; Dasari and Tchounwou, 2014; Rossi and Di Maio, 2016). Although the forms of platinum agents vary when it comes to treatment for specific cancer types, their pharmacological mechanisms are concentrated on inducing DNA damage and cell apoptosis (Dasari and Tchounwou, 2014). As a response to this extreme external stimulation, cancer cells will exert their full resources to escape and manage to survive the lethal strike, which induces genomic, epigenetic, and functional disorders and results in a platinum-resistant cellular biological phenotype (Fang et al., 2018a; Yu et al., 2020). This corresponds with clinical evidence that chemotherapy-treated patients tend to gradually develop platinum resistance, which leads to unsatisfactory clinical outcomes and survival (Agarwal and Kaye, 2003; Alcindor and Beauger, 2011; Fennell et al., 2016).

Epigenetics play a critical and profound role in the genesis, malignancy, and drug response of cancer by regulating DNA-based biological processes, such as replication, damage repair, and transcription (Dawson and Kouzarides, 2012; Peng and Zhong, 2015; Liu et al., 2018). DNA methylation was the first described and possibly the most extensive characteristic modification in human genetic materials. Accumulating evidence has shown that changes in DNA methylation have the potential to alter the platinum sensitivity of cancer cells and predict the chemotherapeutic response of patients (Vera et al., 2017; Fang et al., 2018a). Although several functional studies on specific methylation sites associated with chemoresistance have been conducted, there is an urgent need for a systematic investigation of DNA methylation affiliated with patients’ platinum response via large-scale screening (Vera et al., 2017; Fang et al., 2018a; Fang et al., 2018b).

The Cancer Genome Atlas (TCGA) project collected the clinical and molecular characteristics of over 11,000 patients across different cancer types, making it the perfect database for analyzing and integrating pan-cancer methylation features of different platinum responses. Fang et al. and Zhu et al. provided a significant grouping method and detailed list of patients who received platinum-based treatment (Fang et al., 2018b; Zhu et al., 2020). This study aimed to provide a systematic landscape of DNA methylation associated with platinum treatment and its potential clinical applications across 13 TCGA cancer types.

## Materials and Methods

### Data Collection and Processing

**FIGURE 1:**
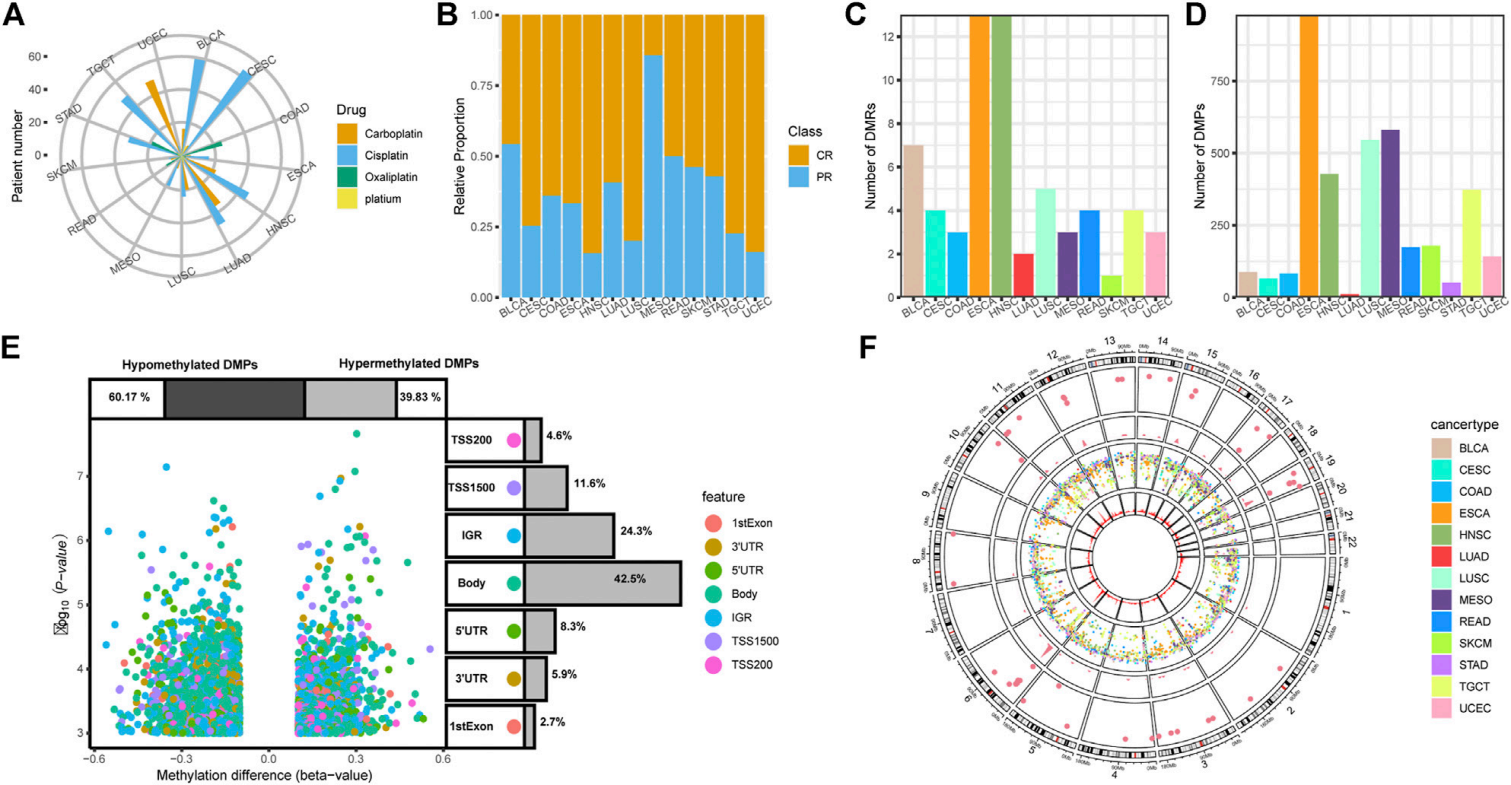
Clinical information and DNA methylation patterns associated with platinum response. **(A)** Clinical application of platinum agents in 13 TCGA cancer types. **(B)** The platinum-sensitive group (CR)/platinum-resistant group (PR) relative proportion of 13 cancer types. **(C)** Number of differentially methylated regions (DMRs) in each cancer type. **(D)** Number of differentially methylated positions (DMPs) in each cancer type. **(E)** Volcano plots of DMPs with annotation of the methylation probe feature. **(F)** Rainfall and density plots of all DMRs and DMPs.

Profiles of 450 K beta value matrixes, transcriptional data and corresponding clinical information across 13 TCGA cancer types were downloaded by “TCGAbiolinks” (Colaprico et al., 2016). Filter, normalization, and annotation of methylation probes were all based on the functions of R package “ChAMP” (Tian et al., 2017). All methylation and transcriptional data were projected and annotated with hg19 (GRCH37). As described previously, we selected patients receiving platinum-based therapy and defined patients of “Complete Response” as platinum-sensitive group (CR) and other descriptions including “Clinical Progressive,” “Stable Disease,” and “Partial Response” as platinum-resistant group (PR) (Zhu et al., 2020). The integrated information of 618 patients with grouping information, cancer type, drug name and response can be seen in Figures 1A,B, and Supplementary Table S1.

Different methylated loci on the overall methylation level and the chromosome level were compared among medians by Wilcoxon rank sum test. Differentially methylated regions (DMRs) were identified by the method of “Bumphunter” and minimum cutoff adjusted *p* value of 0.05 (Jaffe et al., 2012). Differentially methylated positions (DMPs) were defined as *p* value <0.001 and absolute deltaBeta >0.10. Identification of DMRs and DMPs were implemented by functions in “ChAMP”.

### Enrichment Analysis

The bias-free enrichment methods which directly calculate global tests and do gene set enrichment analysis (GSEA) were accomplished by Empirical Bayes gene set enrichment analysis (ebGSEA) method in “ChAMP” package. The results of ebGSEA were evaluated by two main parameters: area under curves (AUCs) from Wilcoxon test and P values detected for each pathway from Wilcoxon test. Gene Ontology (GO) and the Kyoto Encyclopedia of Genes and Genomes (KEGG) enrichment analysis based on corresponding genes of DMPs were conducted by R package “clusterProfiler” (Yu et al., 2012). Reactome pathway analyses were carried out by R package “ReactomePA” (Yu and He, 2016).

### Identification of Transcriptional-Affected DMPs

The corresponding gene names of DMPs were annotated by hg19 and expressions of DMP-related genes were scaled by transcripts per million (TPM) which may be the optimal form of transcriptional normalization. Correlations between methylation and transcriptional data were measured by the Spearman’s test which is not limited to the distribution of data. Transcriptional-affected DMPs should meet the following criteria: 1.) absolute log Fold Change (logFC) of corresponding gene >1; 2.) correlations between values of DMPs and expressions of corresponding genes must be significantly negative (Rho of Spearman’s test < −0.4, adjusted *P* (Benjamini & Hochberg, BH) value of Spearman’s test <0.05).

### Construction of Prognostic and Predictive Models

Screening prognostic DMPs across different cancer types were based on the results of univariate Cox analysis. Schoenfeld tests were conducted and all the Cox models shown in the main text did not violate the proportional hazard assumption. DMPs with adjusted *p* value (BH) < 0.05 were defined as overall survival (OS) related prognostic DMPs. Multivariate Cox analysis of all prognostic DMPs were conducted to adjust for the potential covariates of age and gender (Gender is not a covariate in CESC and UCEC). Kaplan-Meier curves grouped by the value of specific prognostic DMPs were estimated and drawn using R package “survival” and “survminer”.

Identification of platinum-response predictive DMPs were supported by support vector machines (SVMs), which is a binary classifier by maximizing the distance between the classes’ closest points. C-classification method was used to predict the types of CR/PR based on the values of each DMP. Data of every cancer type were randomly split into train sets (2/3 of the total number) and test sets (1/3 of the total number) as described in R-project e1071. Accuracy and F1-measure of test sets by one-time split were used to evaluate predictive models constructed by train sets. AUCs of receiver operator characteristics (ROCs) according to the entire set and the leave-one-out-cross-validation (LOOCV) were used to see the robustness of predictive model. The platinum-response predictive DMPs were supposed to meet the following criteria: 1.) Accuracy >0.8; 2.) F1-measure > 0.8; 3.) AUC of the entire set >0.8; 4.) Mean AUC of LOOCV >0.6. SVMs, ROCs contribution and LOOCV were conducted respectively by R package “e1071,” “pROC,” and “cvms” (Robin et al., 2011).

## Results

### Aberrant DNA Methylation Profiling Patterns Associated With Platinum Chemotherapy Across 13 Human Cancer Types

The platinum agents for chemotherapy and proportions of platinum-sensitive group (CR)/platinum-resistant group (PR) vary according to cancer type, as shown in Figures 1A,B. Cisplatin is the most widely used agent, and oxaliplatin is typically the only choice for gastrointestinal cancers. Carboplatin is used in uterine corpus endometrial carcinoma (UCEC), head-neck squamous cell carcinoma (HNSC), and lung cancers to different extents. There are also a few cases of platinum treatment without specifying agents.

The differences in overall methylation status between the CR and PR groups were observed, and PR groups were found to be in a lower methylation state compared with CR groups in most of tumors, especially in cervical squamous cell carcinoma and endocervical adenocarcinoma (CESC, *p* < 0.05) (Supplementary Figure S1). From the chromosome perspective, 17 types of different methylated chromosomes (*p* < 0.05) also focused on the three cancer types including CESC, esophageal carcinoma (ESCA) and bladder urothelial carcinoma (BLCA). These results of the primary analysis indicated that DNA methylation may play a critical role in the platinum response (Supplementary Figure S2).

We screened 62 DMRs and 3,703 DMPs between CR and PR group across 13 TCGA cancer types. The total number of identified DMRs and DMPs vary by cancer type due to the heterogeneity (Figures 1C,D). ESCA had the highest DMR and DMP values, which correspond with the overall and chromosome level results. Stomach adenocarcinoma (STAD) displayed no DMR and lung adenocarcinoma (LUAD) presented the lowest DMP value, which suggests that there is a weak connection between platinum resistance and DNA methylation in these two cancer types. Volcano plots revealed that 60.17% hypomethylated DMPs of 3,703 overall DMPs are more abundant than 39.83% hypermethylated DMPs, which may indicate that activation of specific genes is more powerful for gaining a platinum-resistant phenotype. Nearly half of the DMP features are gene body. The features of the intergenic region (IGR) and transcription start position (TSS) also take up over 10% (Figure 1E). From the genome perspective, the DMPs of all cancer types are distributed across the whole genome while DMRs are scattered among chromosomes according to rainfall plots. Density plots show that both DMPs and DMRs seem to form peaks on several chromosomes, including the sixth, nineteenth, and eleventh chromosomes, which may indicate the universal significance of methylation on specific genomic regions for platinum response (Figure 1F).

### Cancer Pathways Affected by Methylation and Correlated With Platinum Resistance

**FIGURE 2:**
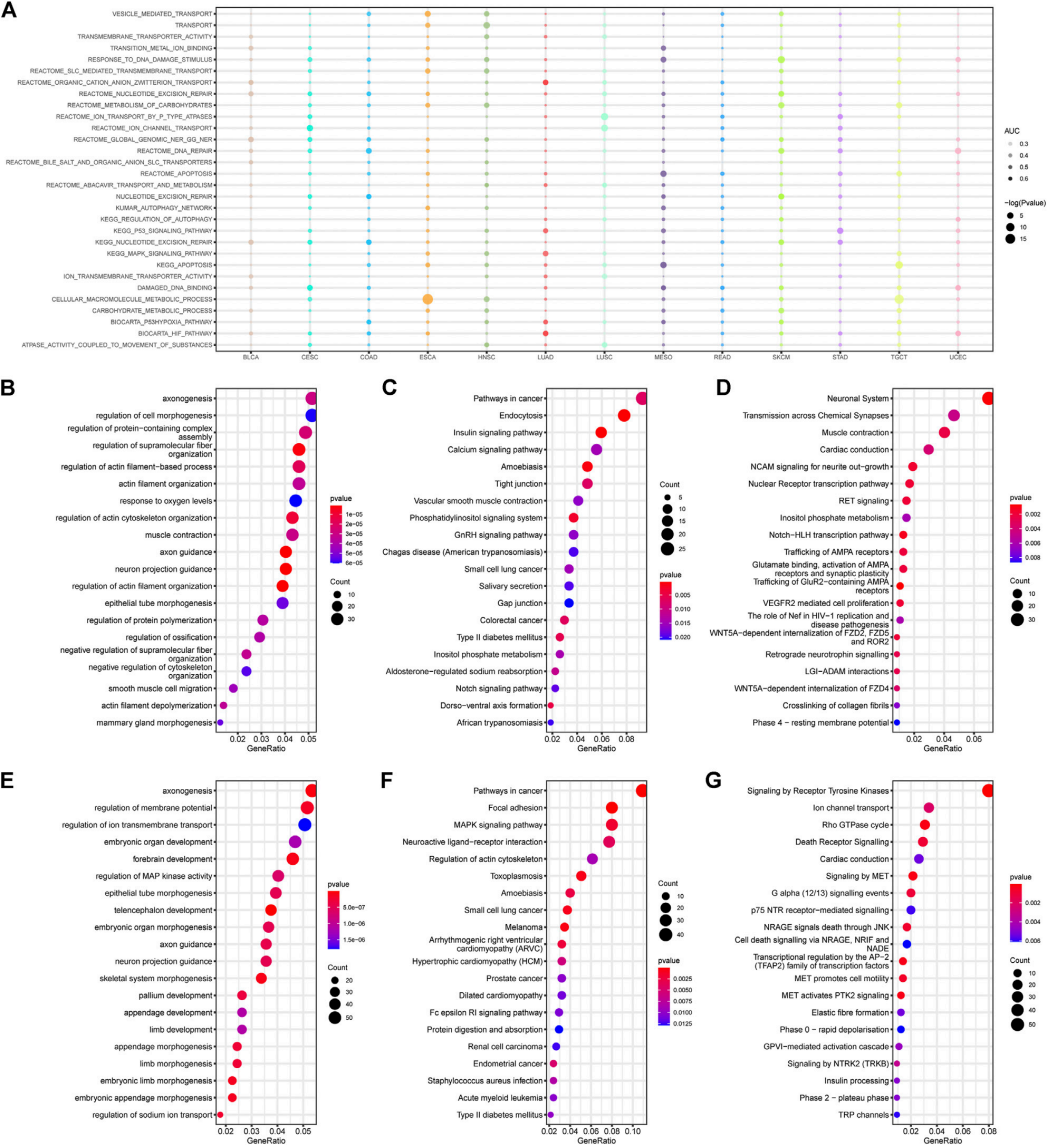
Enrichment results of the methylation profiles correlated with platinum resistance. **(A)** Empirical Bayes gene set enrichment analysis (ebGSEA) results of 30 platinum response-related gene sets across 13 cancer types. **(B–D)** Gene Ontology (GO), the Kyoto Encyclopedia of Genes and Genomes (KEGG), and Reactome enrichment results of the hypermethylated DMPs in PR patients. **(E–G)** GO, KEGG, and Reactome results of the hypomethylated DMPs in PR patients.

Various mechanisms are involved in platinum resistance, including pre-target, on-target, post-target, and off-target resistances (Galluzzi et al., 2012). In order to identify the methylation changes related to the drug response, we selected 30 typical platinum resistance gene sets in the molecular signatures database (MSigDB) and attempted to list the empirical Bayes Gene Set Enrichment Analysis (ebGSEA) results of the CR/PR groups among the 13 cancer types, aiming to obtain an overall landscape of resistance-related enrichment results regardless of DMRs and DMPs (Figure 2A). This shows that all 13 cancer types have significant platinum resistance enrichment on the DNA methylation level, which presents a promising chance to perform enrichment analysis based on DNA methylation data.

Both the hypermethylated and hypomethylated top GO results showed gene sets related to biological axon genesis, morphogenesis, and body development, indicating that some development-related genes may be involved in platinum response (Figures 2B,E). The KEGG results focused on cancer-related enrichment, including pathways in colorectal cancer, melanoma, prostate cancer, and renal cell carcinoma, which suggests that platinum-related phenotypes may be influenced by cancerous genes (Figures 2C,F). The Reactome results revealed hypermethylation of the neuronal system, transmission across chemical synapses, muscle contraction, and hypomethylation of ion channel transport, the Rho GTPase cycle, and death receptor signaling (Figures 2D,G). Generally speaking, pathways including the mitogen-activated protein kinase (MAPK) signaling, inositol phosphate metabolism, and neural genesis-related pathways, appeared more than twice in different enrichment results.

### Methylation and Transcription Characteristics of the Platinum Response in DMRs

To further investigate the common characteristics of DMRs, we downloaded the cytoband annotation of hg19 and projected DMRs onto the cytoband of each chromosome. Both q13.12 on the nineteenth chromosome and p22.1 on the sixth chromosome, which are the most frequent cytobands, had 6 DMRs (Supplementary Table S2). For p22.1 on the sixth chromosome, all DMRs were concentrated in the IGR genome region and did not correspond with any specific gene, suggesting that this region may affect the platinum response in a more complicated way rather than via transcription.

**FIGURE 3:**
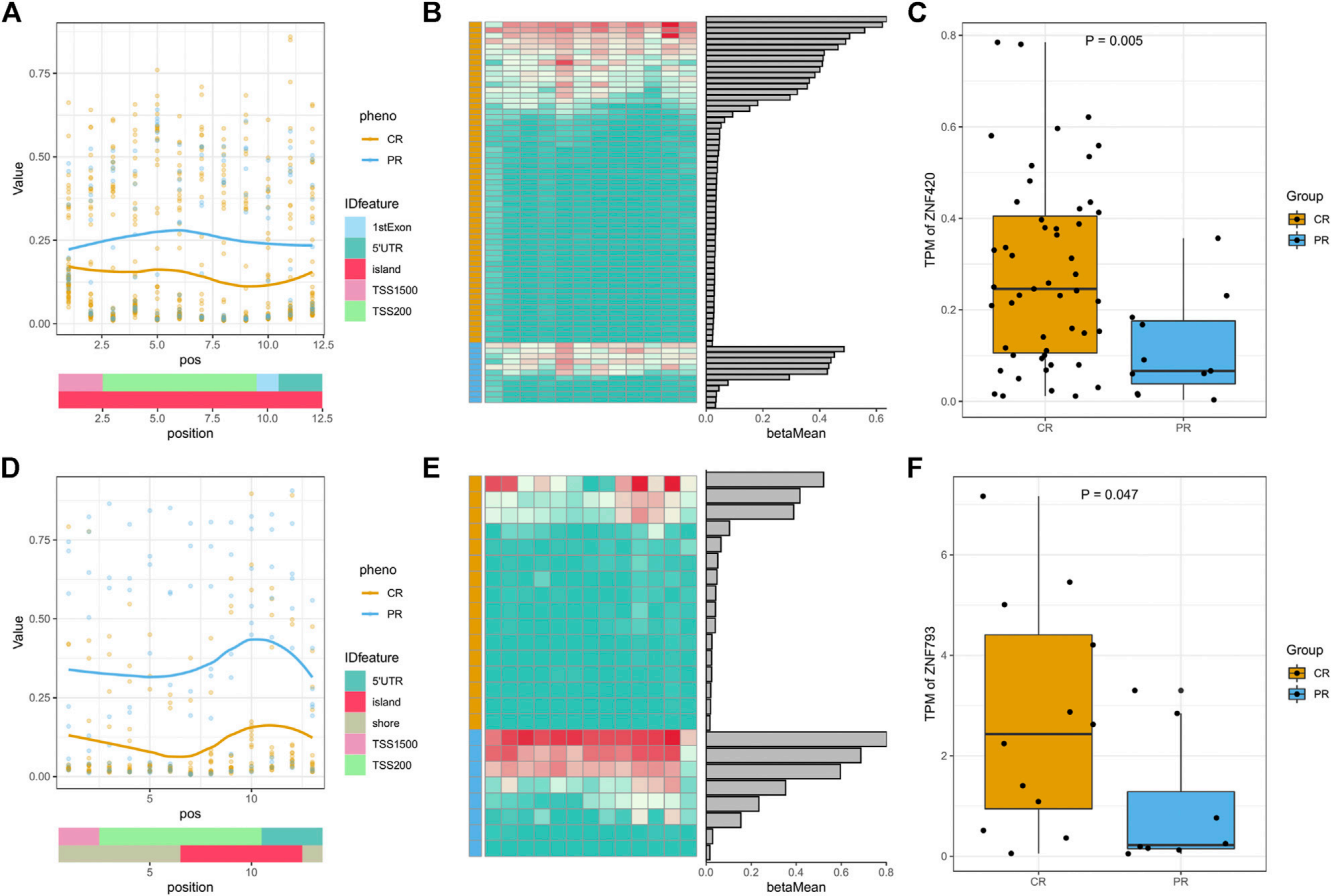
Methylation and expression of ZNF family genes in the DMRs of HNSC and ESCA. **(A)** DMR plot of the methylation status of CR/PR patients with gene feature and CpG islands (CGI) information in head-neck squamous cell carcinoma (HNSC) DMR-8. **(B)** Heatmap of each DMP beta value and bar plots of the mean beta values in HNSC DMR-8. **(C)** Expression (TPM) of relevant genes in specific DMRs between the CR/PR groups in HNSC DMR-8. **(D–F)** DMR plot, heatmap, and gene expression in esophageal carcinoma (ESCA) DMR-8. *p* values were calculated using the Wilcoxon test.

For the 6 DMRs on q13.12 of the nineteenth chromosome, the corresponding genes were all focused on the zinc finger protein (ZNF) family. In HNSC, the overall methylation status of PR was higher than that of CR, and the expression of the corresponding ZNF420 gene of PR was lower than that of CR, both of which follow a typical hypermethylation-transcription-repressive model (Figures 3A–C). In ESCA, the hypermethylation of PR and low expression of ZNF493 were more obvious (Figures 3D−F). In rectum adenocarcinoma (READ), the methylation status was the same as in HNSC and ESCA but did not induce significant transcriptional changes (Supplementary Figure S3). In CESC and lung squamous cell carcinoma (LUSC), the methylation value of CR was higher than that of PR, but none of the corresponding ZNF family gene expression levels were significantly different (Supplementary Figure S3). Although ZNF family genes have different methylation and expression patterns in different cancer types, all of the above results indicate the significance of methyl modification of ZNF genes in the platinum response of patients with different cancer types.

### Regulation of DMPs and Expression Profiles in Platinum-Treated Patients

**FIGURE 4:**
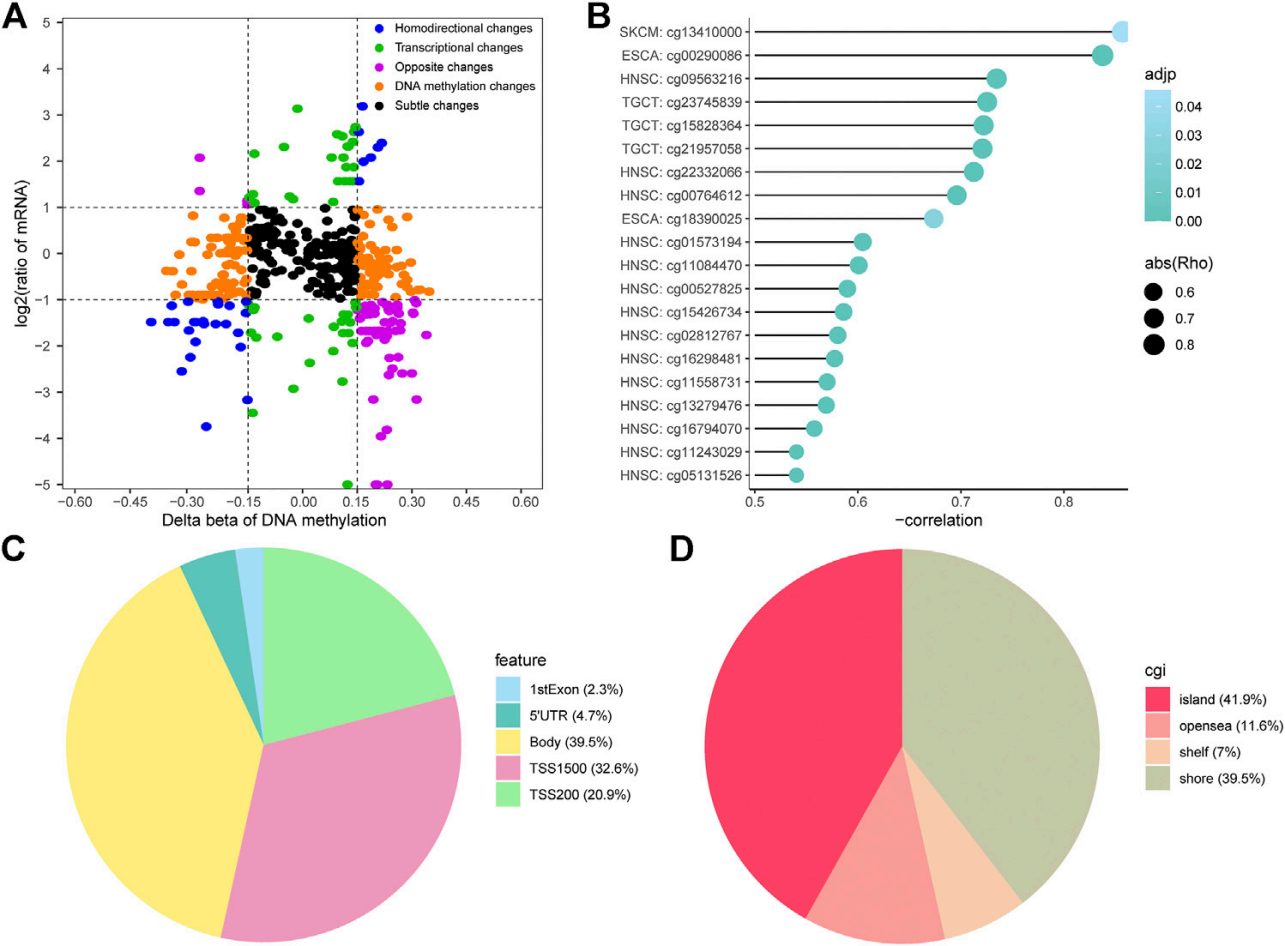
Identification of the methylation-transcription axis in platinum-treated patients. **(A)** Methylation and transcription plots of transcription-regulative DMPs and corresponding genes in the CR/PR groups. **(B)** Top 20 DMPs with negative correlation coefficients in the opposite change fractions. **(C–D)** Proportion of genes and CpG islands (CGI) features of transcription-affective DMPs.

It is a commonly accepted theory that hypermethylation or hypomethylation of DNA will induce silencing or activation of corresponding gene transcription, especially for CpG islands. We screened all DMPs using the Spearman correlation test criteria described in the Materials and methods and divided them into five different regions based on the delta beta probe values of CR/PR and log fold-change (FC) of corresponding mRNA, including transcriptional changes, DNA methylation changes, homodirectional changes, opposite changes, and subtle changes (Figure 4A). According to the methylation-transcription theory, we focused on the probes and genes with opposite changes.

To better demonstrate the relationship between DMPs and gene expression, we drew a lollipop plot of minus correlation coefficient Rho and P values for the top 20 probes ranked by absolute Rho (Figure 4B). There were 43 DMPs negatively associated with the expression of 28 genes (Supplementary Table S3). The proportions of DMPs in gene bodies, TSS1500, and TSS200 were 39.5, 32.6, and 20.9%, respectively, confirming their indispensable roles in transcription (Figure 4C). Features including islands and shores respectively accounted for 41.9 and 39.5% of the overall CpG islands (CGI) feature, respectively, which emphasizes the significance of the CpG theory (Figure 4D). As seen in Supplementary Table S3, there were 26 hypermethylated transcription-repressive genes and two hypomethylated transcription-activated genes. Hypermethylated deactivated pathways included negative regulation of protein phosphorylation, signaling by Hedgehog, and tight junctions. Hypomethylated activated pathways consisted of DNA damage-related pathways, the cellular response to hydroxyurea pathway, and metabolism-related pathways. These enrichment results share some of the same or similar gene sets as those in Figure 2, including tight junctions, DNA damage-related pathways, and metabolism-related pathways (Supplementary Figure S4), which indicates the significance of epigenetic-functional pathways in platinum resistance. Our data suggest the possibility of significant methylation-transcription connections of the genes in platinum-treated patients with different cancer types.

### DMP Patterns Associated With Clinical Prognosis and Platinum Response

**FIGURE 5:**
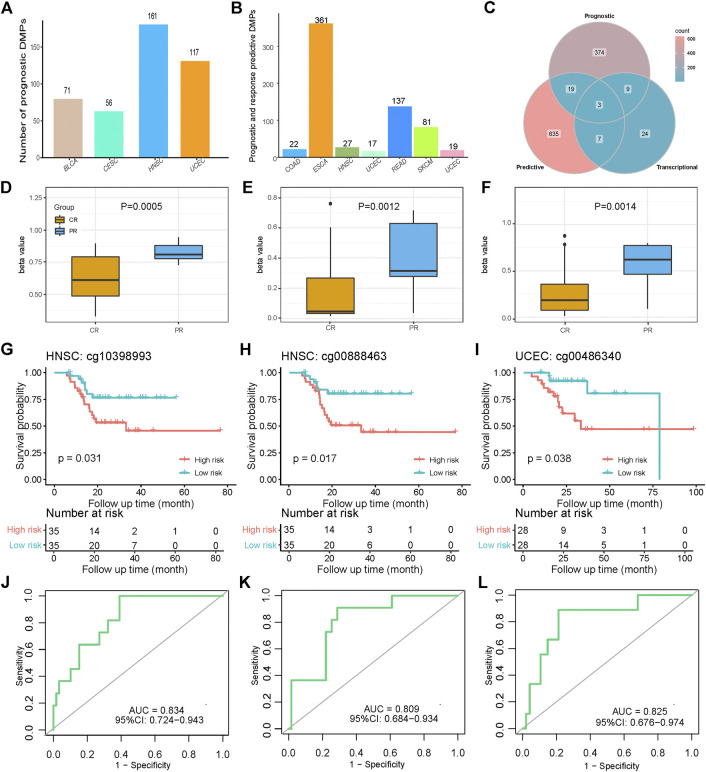
Prognostic and platinum response-predictive DMP models. **(A)** Distribution of 405 prognostic DMPs among four cancer types. **(B)** Distribution of 664 platinum response-predictive DMPs among eight cancer types. **(C)** Intersection Venn plot of the prognostic, predictive, and transcriptional DMPs. **(D–F)** Different beta values of cg10398993, and cg00888463 in HNSC as well as cg00486340 in UCEC between the CR and PR groups. *p* values were calculated using the Wilcoxon test. **(G–I)** K-M survival plots grouped by the beta values of the three DMPs. *p* values were directly calculated using log-rank test. **(J–L)** Receiver operator characteristics (ROC) curves of the three DMPs.

Patient survival is one of the most important indications for assessing platinum-based chemotherapy responses. Since it is limited to the number of patients in each cancer type, overall survival (OS) is the most common and easiest available index of patient survival. There were 405 prognostic DMPs that could serve as risk factors and predictors of patient OS in four cancer types after univariate Cox screening (Supplementary Table S4). P values of over 99% (404/405) of the prognostic DMPs were less than 0.10 after adjusting for other potential clinical covariates using multivariate Cox analysis (Supplementary Table S4). HNSC, UCEC, BLCA, and CESC had more than 50 prognostic DMPs, which suggests that DMP values in these four cancer types can more easily play the role of survival-risk factors for OS in platinum-treated patients (Figure 5A).

Clinically, selection of treatment mainly depends on how the previous patients responded to various treatments. For chemotherapy patients, there is a strong need for valid biomarkers that can predict and assess the reaction of platinum drugs. SVMs provide an optimal method for identifying DMPs that are able to classify the CR and PR classes. As described previously, we defined response-predictive DMPs as potential biomarkers for classifying platinum response groups with promising accuracy, F1 measure, and the AUCs of ROCs. There were a total of 664 response-predictive DMPs distributed in eight cancer types, including COAD, ESCA, HNSC, LUSC, READ, SKCM, and UCEC (Supplementary Table S5; Figure 5B).

There were 12 potentially transcription-regulative DMPs that impacted the survival of patients treated with platinum-based methods (Figure 5C). Interestingly, there were three identified prognostic risk DMPs that also acted as response-predictive DMPs, including cg10398993, and cg00888463 in HNSC as well as cg00486340 in UCEC (Figure 5C). Coincidentally, they were all hypermethylated in PR patients and therefore followed the hypermethylation-deactivation patterns in platinum-treated samples. The independent P values of the Wilcoxon test were 0.0005, 0.0012, and 0.0014, respectively, which indicated significant differences in methylation (Figures 5D−F). The corresponding genes of these three DMPs were mesothelin (MSLN), protein kinase cAMP-dependent type II regulatory subunit beta (PRKAR2B), and msh homeobox 1 (MSX1), all of which correlated with tumor development, malignancy, and treatment (Yue et al., 2018b; Hilliard, 2018; Sha et al., 2018). Patients with hypermethylated status of these three genes were considered as the high-risk group and had poorer survival than those with hypomethylated status. The log-rank P values were calculated to be 0.031, 0.017, and 0.038, respectively, which showed well-performed prognostic distinctions between platinum-treated groups based on the specific methylation status of these four genes (Figures 5G–I). The AUCs of the ROC curves of the three DMPs were 0.834, 0.809, and 0.825, respectively, and all of the lower values of the 95% confidence intervals (CIs) were far greater than 0.5, which indicates promising predictive functions of the platinum-based chemotherapy response (Figures 5J–L). The mean AUCs of LOOCV of the three DMPs were 0.846, 0.627, and 0.780, respectively, which shows the predictive robustness of these three DMPs (Supplementary Table S5). This analysis emphasizes the potential biological and clinical significance of the methylation of these genes for platinum resistance in cancer.

**FIGURE 6:**
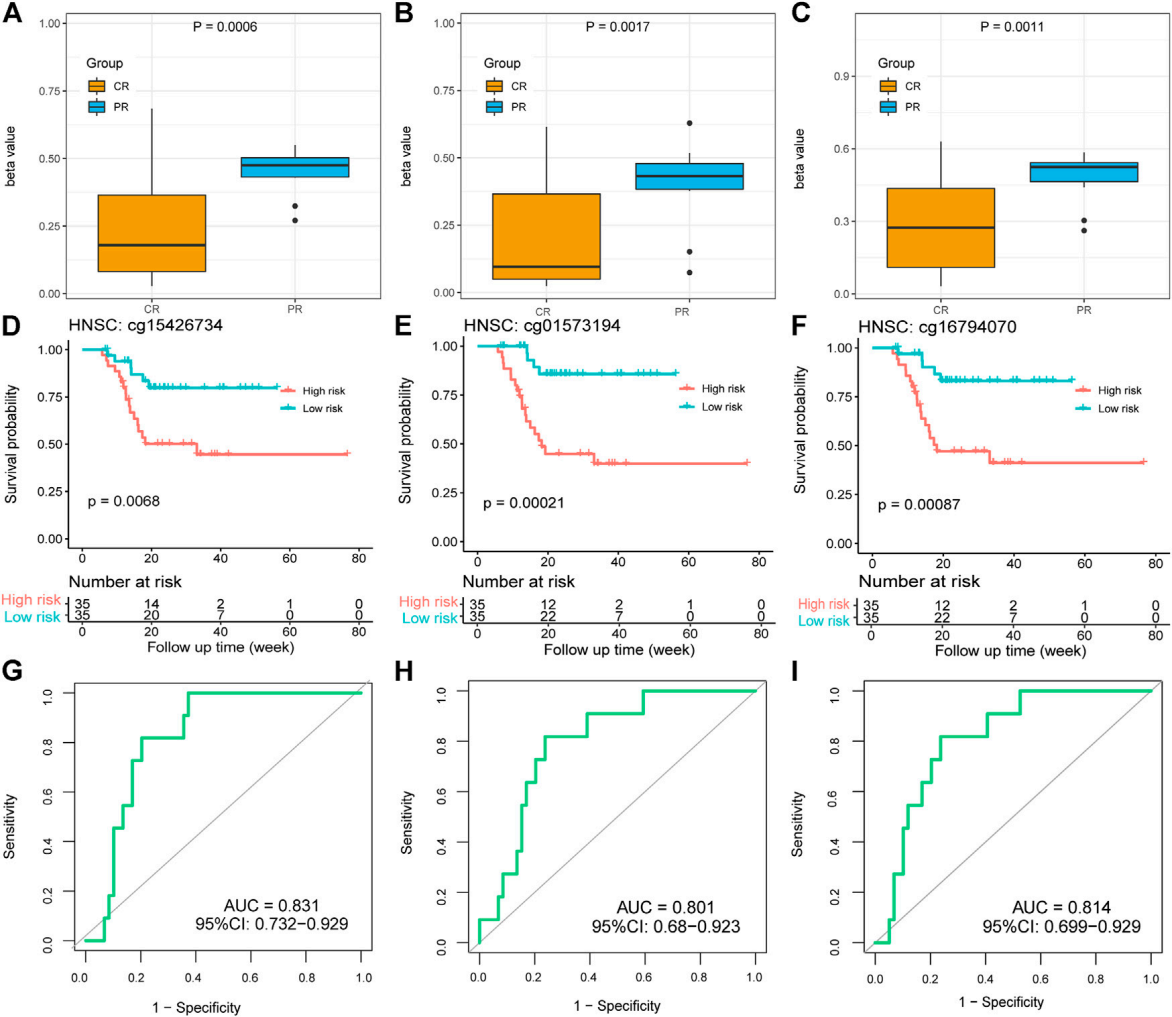
Methylation and clinical values of PARD6A in platinum-treated patients with HNSC. **(A–C)** Different beta values of cg15426734, cg01573194, and cg16794070 between the CR and PR groups in HNSC. *p* values were calculated using the Wilcoxon test. **(D–F)** K-M survival plots grouped by the beta values of cg15426734, cg01573194, and cg16794070 in HNSC. *p* values were directly calculated using log-rank test. **(G–I)** ROC curves of the three DMPs.

It is worth mentioning that there were three methylation probes (cg15426734, cg01573194, and cg16794070) of the par-6 family cell polarity regulator alpha (PARD6A) significantly hypermethylated in the HNSC PR groups, which was proven by the *p* values of 0.0006, 0.0017, and 0.0011, respectively (Figure 6A–C). High-risk patients with hypermethylation showed poorer OS than low-risk patients with hypomethylation. The log-rank *p* values were 0.0068, 0.00021, and 0.00087, respectively, which revealed significant survival differences between the risk groups based on the values of the three PARD6A probes (Figure 6D–F). Although the three PARD6A probes were not defined as response-predictive DMPs because of their slightly inferior accuracy (0.783) and mean AUCs of LOOCV (0.716, 0.474, and 0.706), the AUCs of these entire sets were all over 0.8, which still indicate potential well-performed response-predictive functions (Figure 6G-I). Thus, our investigation suggests a novel clinical and biological understanding of PARD6A methylation.

## Discussion

Platinum-based chemotherapy resistance has always been an intractable problem for oncology clinicians. TCGA provides a landmark platform for analyzing the epigenomic data related to chemotherapy, and several studies have focused on the role of a specific gene or site in chemoresistance for a specific cancer type (Grasse et al., 2018; Mase et al., 2019; Wang et al., 2019). In recent years, the importance of pharmacological epigenetics has received increasing attention from medical researchers and clinicians. DMRs and DMPs are worthwhile indicators to determine the molecular characteristics (Choudhury et al., 2020; Hernandez-Meza et al., 2020). In this study, we attempted to analyze the methylation profiles at various levels from the overall methylation status to the value of a single probe by integrating 618 platinum-treated patients across 13 TCGA cancer types. We screened 62 DMRs and 3,703 DMPs between CR and PR group, which included 2,232 hypomethylated DMPs and 1,471 hypermethylated DMPs. Enrichment results revealed methylation changes in platinum resistance-related pathways, though 13 cancer type had different outstanding enrichment results. Pathways including metabolism-related pathways, MAPK signaling, and neural genesis-related pathways might be epigenetically significant associated with platinum resistance. MAPK signaling is significant in ovarian patients with progression free survival over 6 months under platinum treatment (Matei et al., 2012). Inhibitors toward MAPK signaling are able to enhance sensitivity to platinum agents in melanoma cells (Makino et al., 2018). The role of MAPK signaling, which is specifically activated in platinum-based therapy, is biologically and clinically significant (Hernandez Losa et al., 2003; Makino et al., 2018). Our results indicate the activation of MAPK signaling under platinum-treatment might be driven by DNA hypomethylation of genes in MAPK pathway.

Corresponding transcriptional changes may be the most widely accepted biological mechanism of platinum response-related DNA methylation. Here, we identified the methylation and transcription characteristics of platinum response in DMRs. Leaving intergenic regions alone, q13.12 of the nineteenth chromosome is the most interesting cytoband and their corresponding genes of ZNF family might have extensive methylation patterns and potential biological significance across various cancer types. Recently, Mishra et al. revealed that methylation of ZNF genes could serve as a molecular clustering basis for pancreatic cancer (Mishra and Guda, 2017). A few analyses have focused on the clinical significance of ZNF genes’ expression on drug resistance (Lee et al., 2013; Marzbany et al., 2019). Several functional studies on the role of mediating chemoresistance point out that ZNF genes may induce chemoresistance via DNA repair and activation of resistance genes’ transcription (Rink et al., 2013; Chen et al., 2018). Our study of DMRs suggests that methylation and expression of ZNF genes may be associated with platinum resistance in multiple cancers.

As for potential transcription-affective abilities of DMPs, we integrated methylation and transcription profiles and identified the typical genes/probes following widely known opposite directional patterns. We identified 26 hypermethylated transcription-repressive genes with 41 DMPs and two hypomethylated transcription-active genes with two DMPs in the profiles of platinum-resistant patients, which confirmed the existence and significance of the methylation-transcription axis in platinum response. These genes in methylation-transcription axis under platinum treatment might play a significant and functional role in platinum resistance, some of which has been reported. Overexpression of BLM RecQ like helicase (BLM) is able to induce DNA damage and increase sensitivity to platinum agents in triple-negative breast cancer and ovarian cancer (Birkbak et al., 2018). Overexpressed cyclin D1 (CCND1) can hyperactivate cyclin-dependent kinase 4 and 6 (CDK4/6) and induce cisplatin resistance in HNSC, TGCT and other cancers (Noel et al., 2010; Adkins et al., 2019). Our results might provide an interesting list of genes which participate in platinum resistance through methylation-transcription patterns.

DMPs are promising for identifying potential biological and clinical biomarkers. Regarding two of the most interesting questions, patient prognosis and response to platinum, we constructed models of 405 prognostic and 664 platinum response-predictive DMPs associated with platinum-based chemotherapy, which established a novel perspective of guiding clinical selection of optimal treatment based on pan-cancer methylation profiles. More importantly, six DMPs of four genes may have transcription-affected, prognostic, and platinum-response predictive functions in HNSC and UCEC, including MSLN (cg10398993), PRKAR2B (cg00888463), MSX1 (cg00486340), and PARD6A (cg15426734, cg01573194, and cg16794070). MSLN, a glycoprotein on the cell surface, is overexpressed in many cancers and is relevant to novel anti-cancer treatment by regulating the tumor microenvironment (Hilliard, 2018; Cerise et al., 2019). PRKAR2B plays a significant oncogenic and metastasis-promoting role in prostate cancer, especially in castration-resistant prostate cancers (Sha et al., 2018; Xia et al., 2020). MSX1 is associated with various malignant cancers and is frequently methylated in cervical and breast cancer (Yue et al., 2018a; Yue et al., 2018b). It should be pointed out that there are three PARD6A probes serving as survival risk factors with potential biological, prognostic, and response-predictive values in HNSC. PARD6A is a cell membrane protein that plays a pivotal role in enhancing the migration, invasion, and proliferation of cancer cells (Ruan et al., 2017). Methylation status and potential clinical values of PARD6A should capture the attention of researchers working on HNSC-related biomarkers. Our insufficient analytical methods might be a few minor flaws when identifying prognostic and platinum response-predictive DMPs. Other potential clinical covariates should be adjusted for further prognostic analysis and robustness of the response-predictive DMPs should be proved and verified in other external datasets. Anyway, our work provided a number of DMPs as potential biomarkers and candidates of further research. These five genes and methylation status may be promising candidates for further biological experiments and clinical studies.

Collectively, through systematic DNA methylation array analysis of 618 platinum-treated patients across 13 TCGA cancer types, we screened 62 DMRs and 3,703 DMPs between different responses to platinum-based chemotherapy. We constructed models of 405 prognostic and 664 platinum response-predictive DMPs associated with platinum-based chemotherapy, which may potentially help to guide the identification of additional clinical and epigenetic biomarkers as well as novel designs for molecular biological experiments related to platinum resistance.

## Data Availability

The original contributions presented in the study are included in the article/Supplementary Material, further inquiries can be directed to the corresponding author.
