# Supplementary material for: Systematic Investigation of DNA Methylation Associated With Platinum Chemotherapy Resistance Across 13 Cancer Types
Source: Front Pharmacol. 2021 Apr 29;12:616529. doi: 10.3389/fphar.2021.616529 (PMC8117351; doi:10.3389/fphar.2021.616529)
Supplement: Supplementary file 1 [file DataSheet2.PDF]

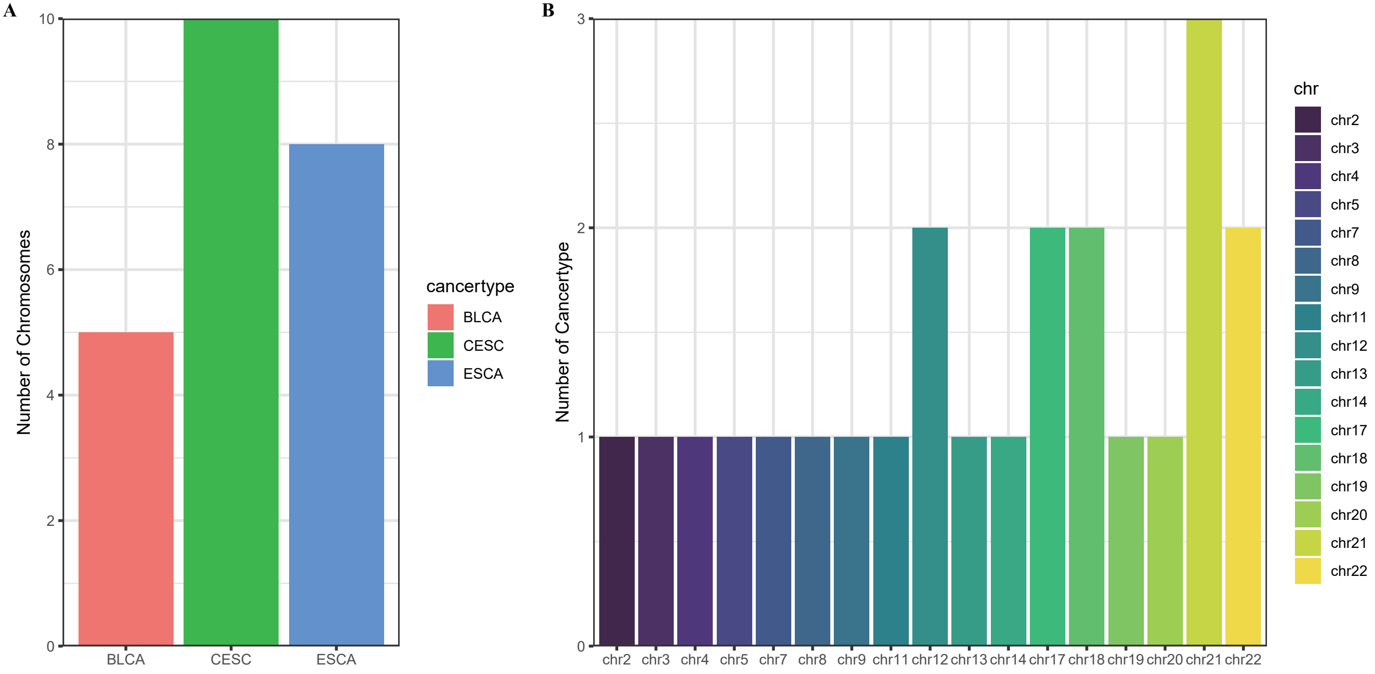

FigureS2: Distribution of differentially methylation chromosomes between CR and PR. The distribution of differentially methylated chromosomes among cancer types (A) and among chromosomes (B). Differentially methylated chromosomes were defined as the P value by Wilcoxon test in comparison of the mean value on chromosome levels between CR and PR < 0·05.
the P value by Wilcoxon test in comparison of the mean value on chromosome levels between CR and PR < 0.05.
