# Supplementary figures and images for: Systematic Investigation of DNA Methylation Associated With Platinum Chemotherapy Resistance Across 13 Cancer Types
Source: Front Pharmacol. 2021 Apr 29;12:616529. doi: 10.3389/fphar.2021.616529 (PMC8117351; doi:10.3389/fphar.2021.616529)

a

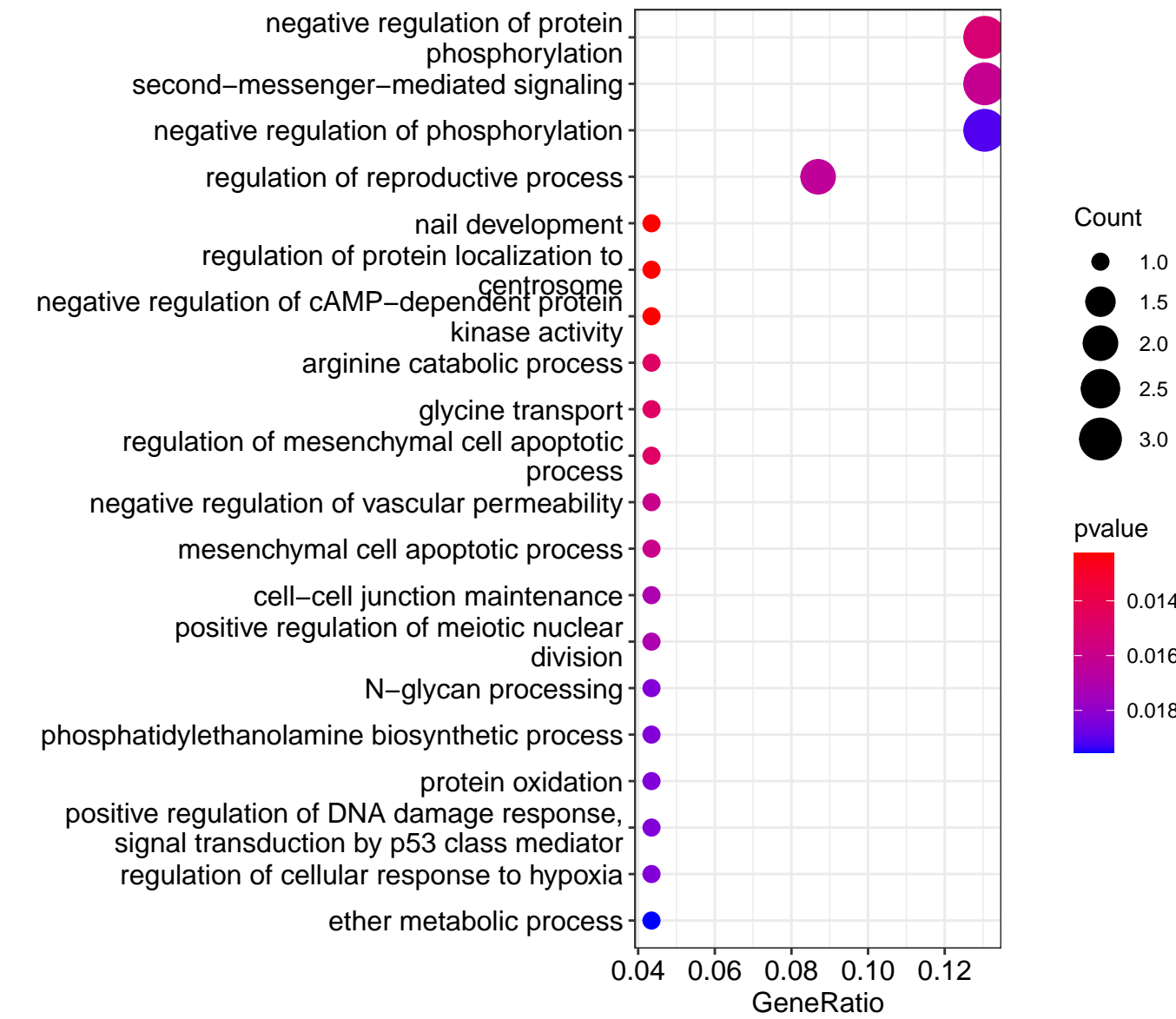

b

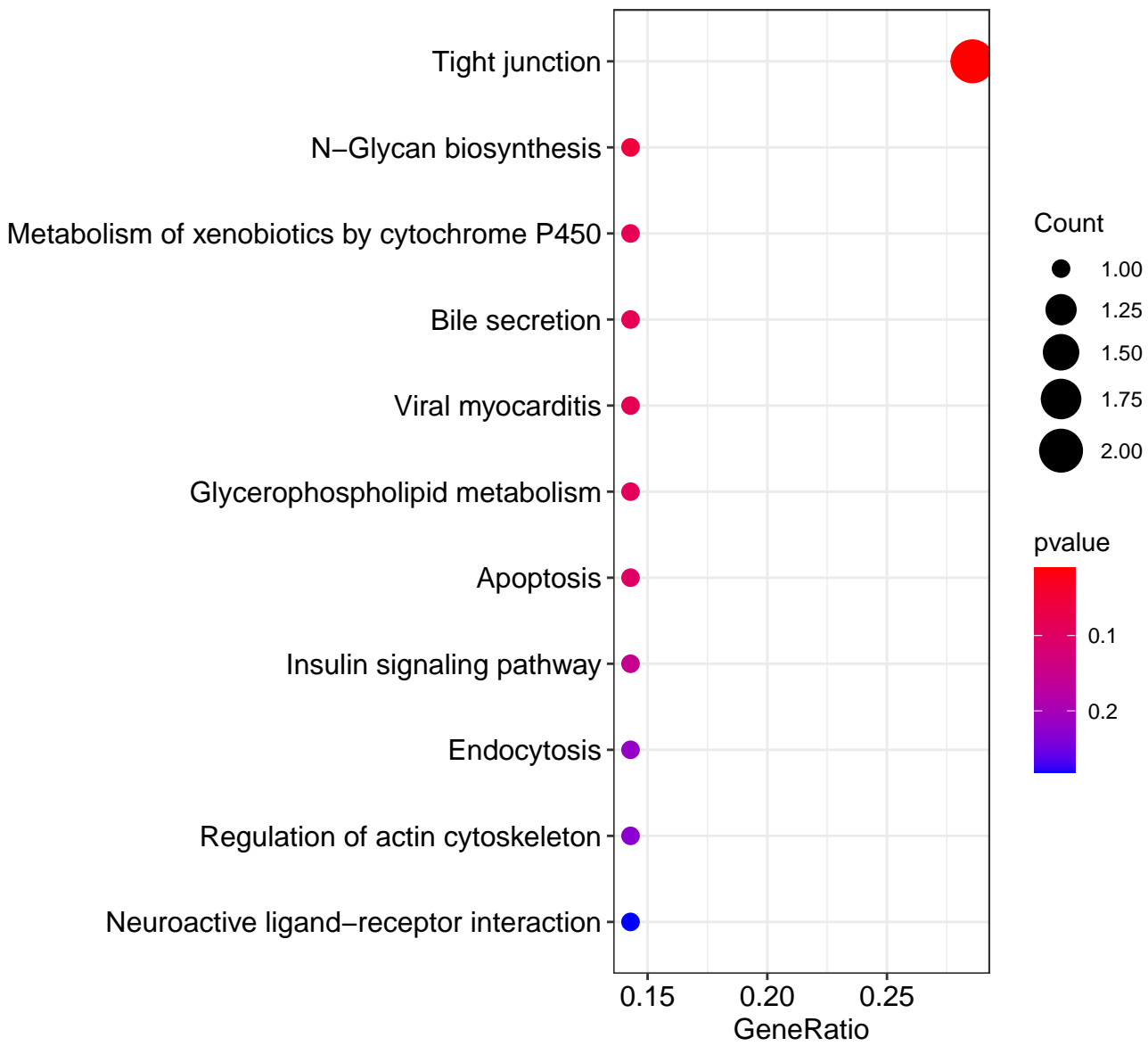

c

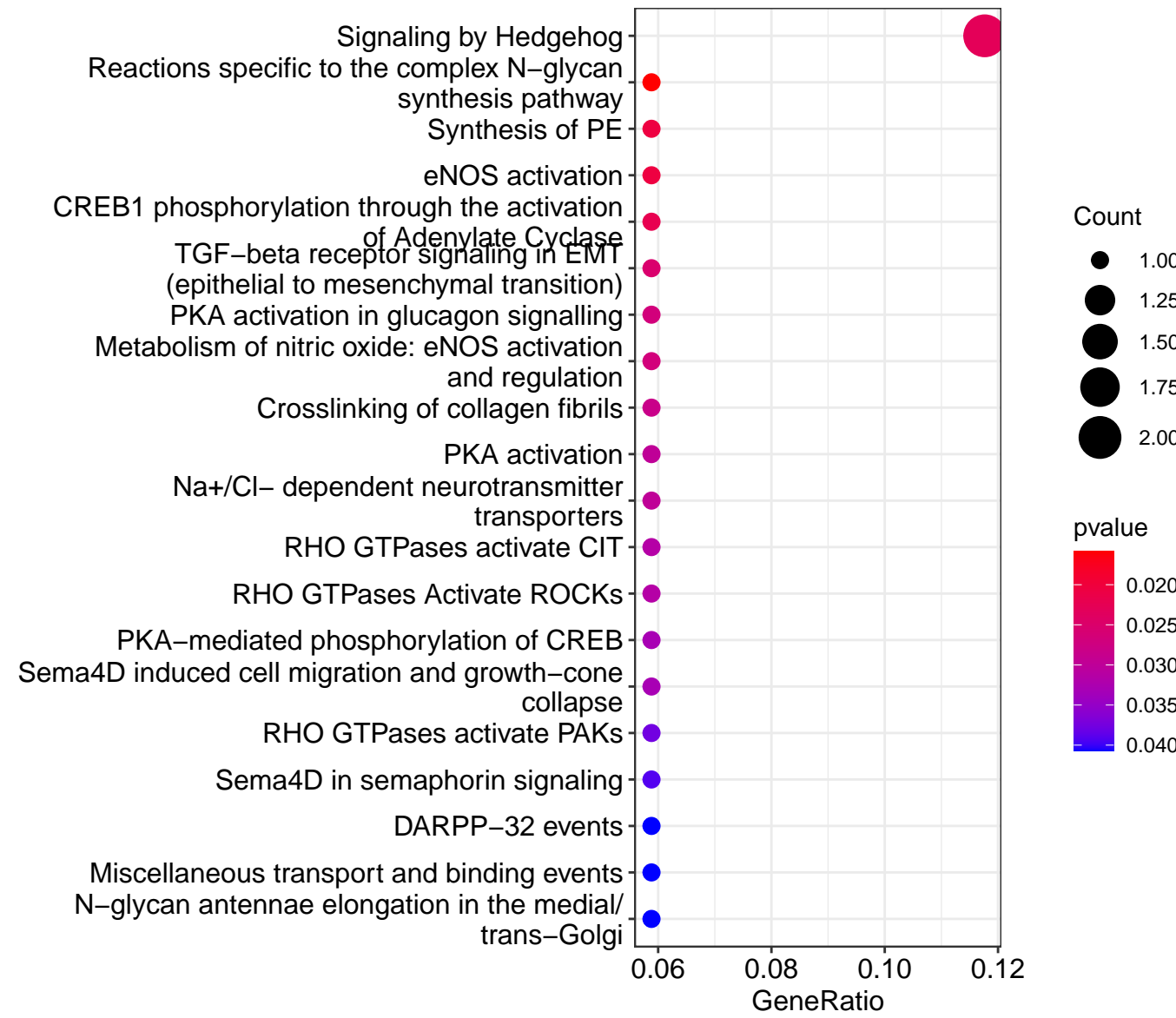

d

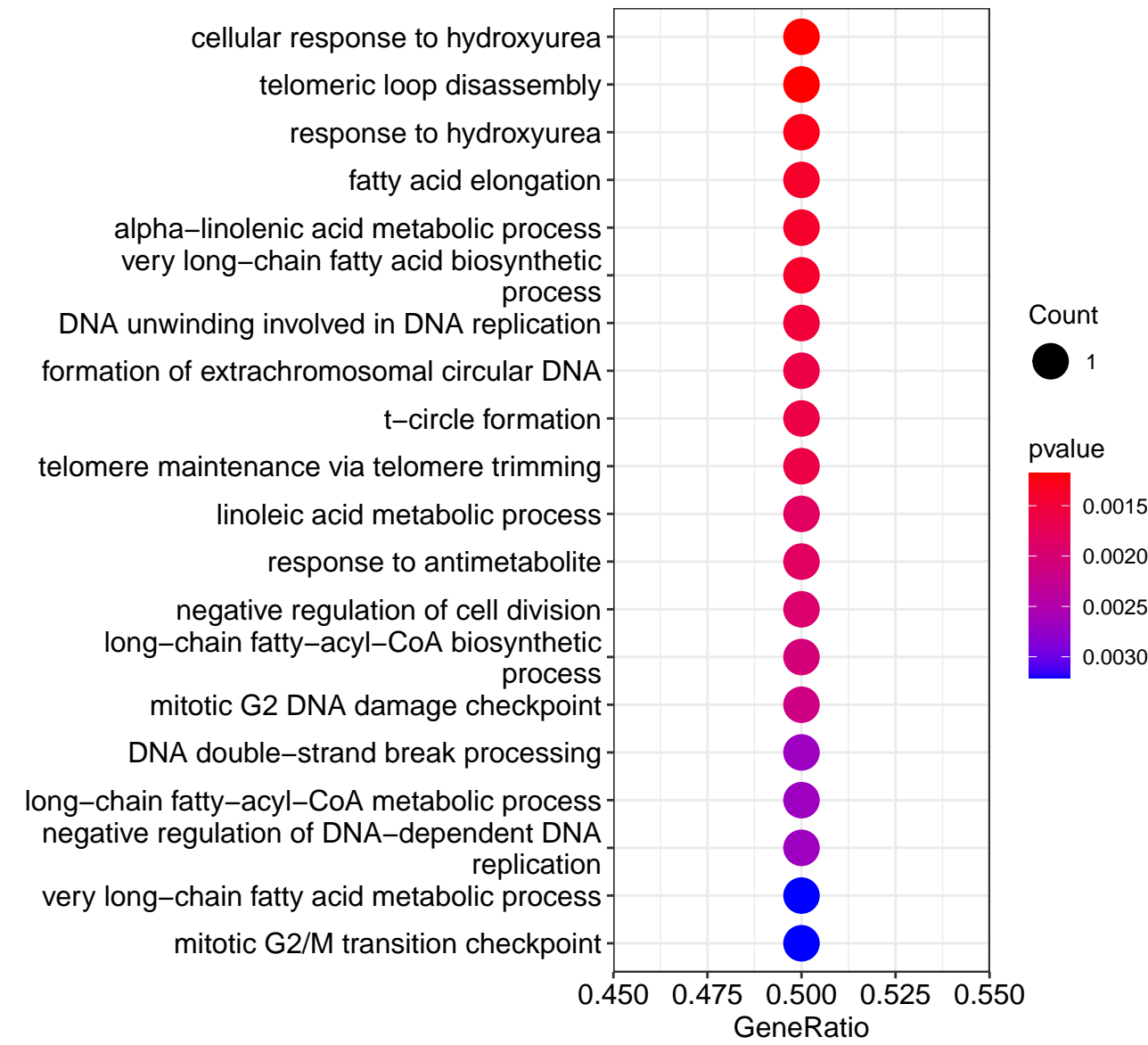

e

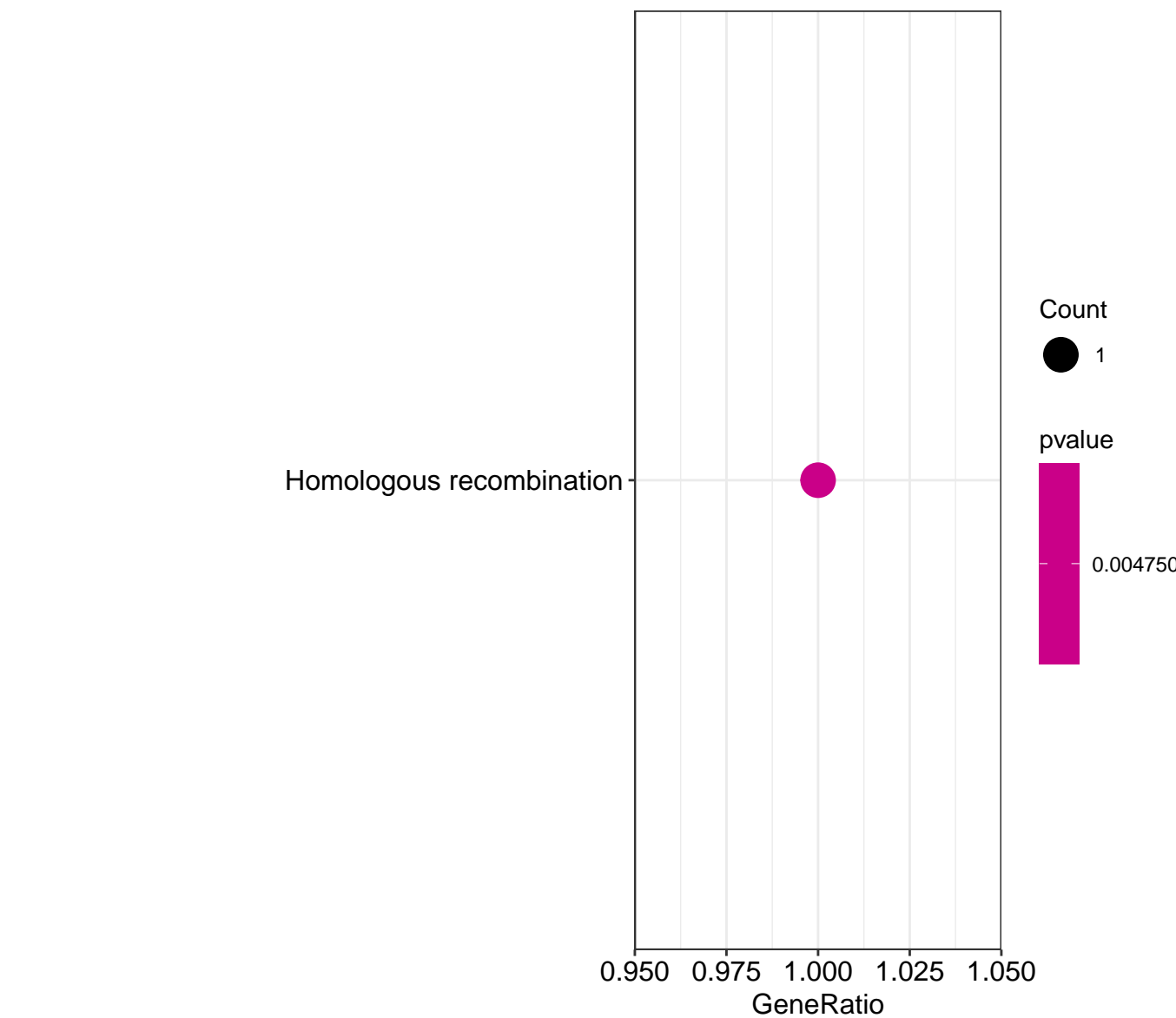

f

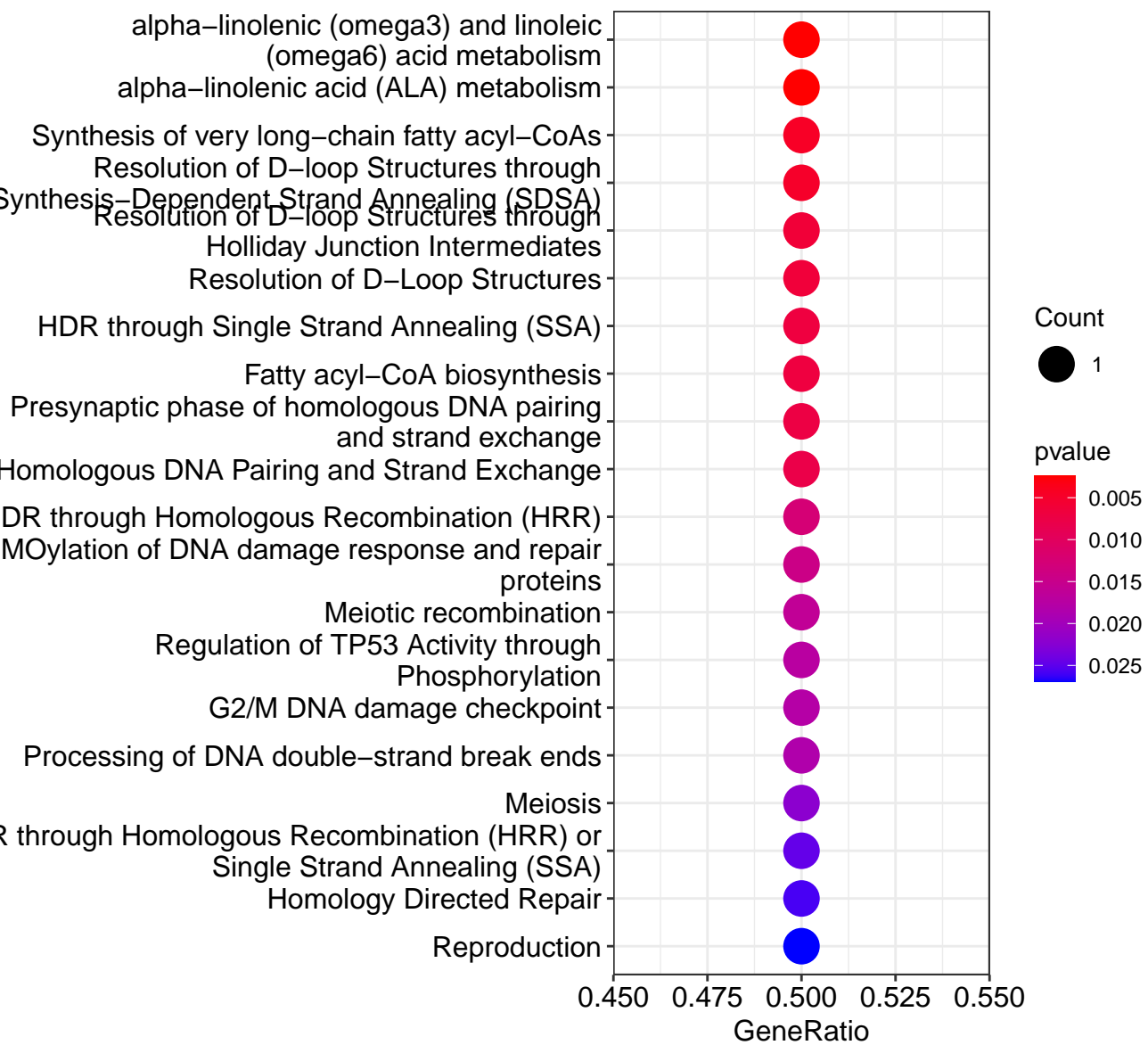

Supplement: Supplementary file 3 [file DataSheet4.PDF]

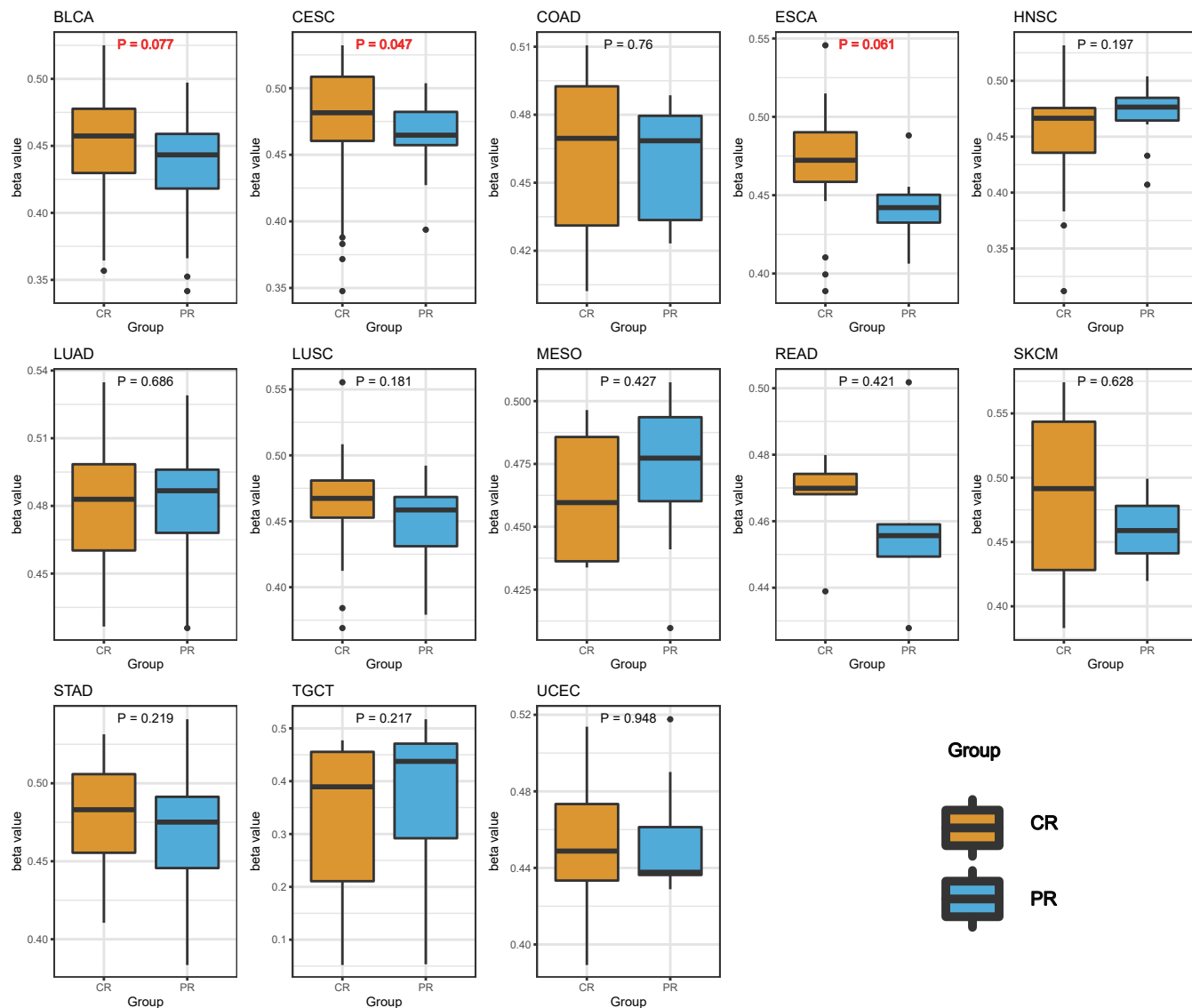

**FigureS1:** The overall methylation status between CR and PR groups in 13 different cancer types.

Supplement: Supplementary file 6 [file DataSheet1.PDF]
