# Supplementary material for: Systematic Investigation of DNA Methylation Associated With Platinum Chemotherapy Resistance Across 13 Cancer Types
Source: Front Pharmacol. 2021 Apr 29;12:616529. doi: 10.3389/fphar.2021.616529 (PMC8117351; doi:10.3389/fphar.2021.616529)
Supplement: Supplementary file 5 [file DataSheet3.PDF]

CESC DMR-2

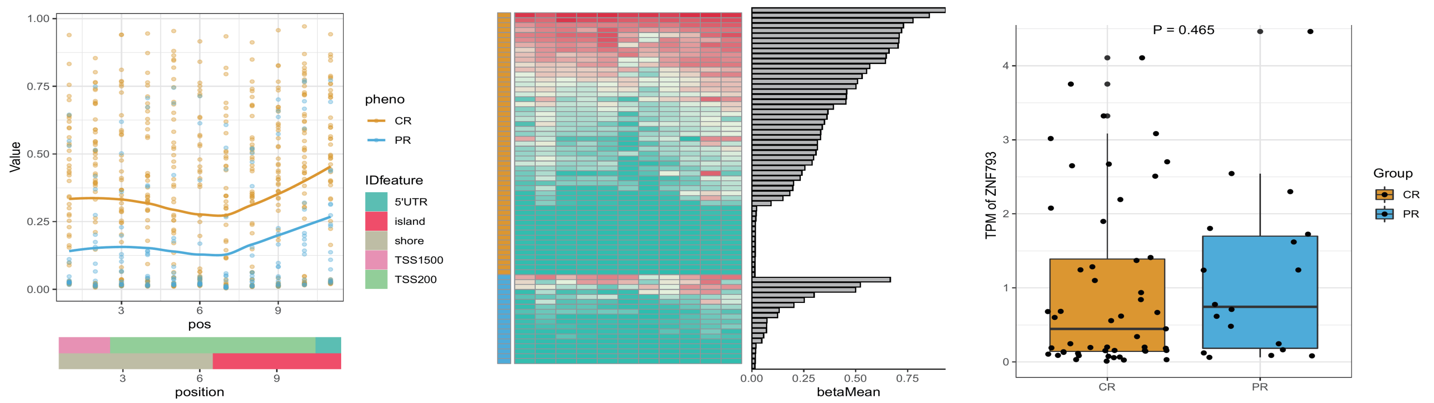

CESC DMR-3

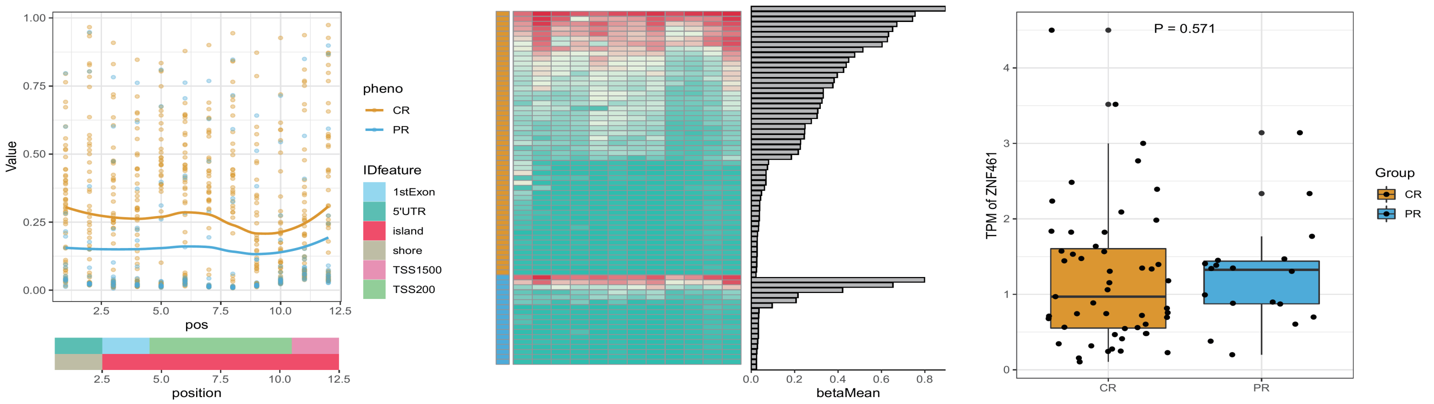

LUAD DMR-5

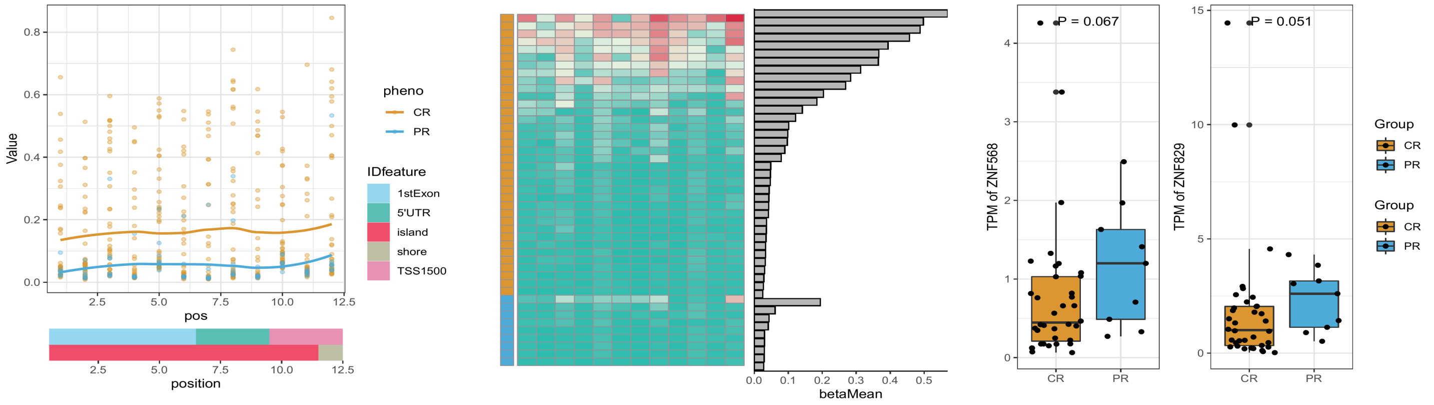

READ DMR-2

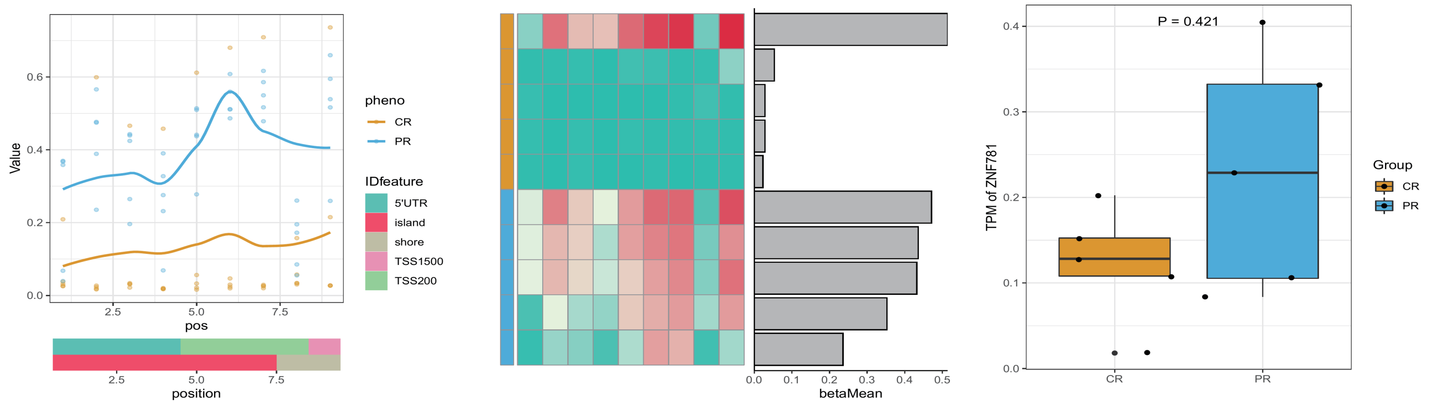

FigureS3: Methylation and expression of ZNF family genes in DMR of CESC, LUAD, and READ. The DMR plot, volcano (and expression boxplot of corresponding gene) of CESC DMR-2, CESC DMR-3, LUAD DMR-5, and READ DMR-2.
